# Primordial germ cell-like cells residing in the pituitary may serve as the origin of intracranial germ cell tumors

**DOI:** 10.1038/s41598-026-38060-2

**Published:** 2026-02-03

**Authors:** Yi Zhang, Lei Zhang, Zhengyang Shen, Jifang Liu, Zhang Ye, Kan Deng, Yong Yao, Daishu Han

**Affiliations:** 1 Department of Neurosurgery, Peking Union Medical College Hospital, Chinese Academy of Medical Science and Peking Union Medical College, Beijing, China; 2 Institute of Basic Medical Sciences, Chinese Academy of Medical Sciences, School of Basic Medicine, Peking Union Medical College, Beijing, China; 3 Department of Traditional Chinese Medicine, The First People Hospital of Lianyungang City, Jiangsu, China

**Keywords:** Intracranial germ cell tumors, Primordial germ cell-like cells, Pituitary gland, Germ cell markers, Tumorigenesis, Cancer, Cell biology, Developmental biology, Stem cells

## Abstract

Intracranial germ cell tumors (iGCTs) are rare, histologically classified as extragonadal germ cell tumors, and predominantly affect children and adolescents. These tumors are commonly found in the midline of the brain, with a notable male predominance and varying geographic incidences. The origins of iGCTs have been debated, particularly whether they arise from abnormal migration of primordial germ cells (PGCs) or transformed embryonic stem cells (ESCs). In this study, we hypothesize the potential presence of PGC-like cells in the pituitary gland, and these cells may differentiate into iGCTs during the complex and frequent regulation of the hypothalamic-pituitary–gonadal axis (HPGA).We analyzed the expression of four germ cell markers—MVH, OCT4, C-kit, and PLZF—using immunohistochemistry, Western blotting, and real-time quantitative PCR in human pituitary tissues, pituitary tumors, and pituitary germ cell tumors. Our findings indicate significant expression of these markers in pituitary tissues, with the highest levels found in pituitary germ cell tumors. These results support the existence of PGC-like cells that within the normal pituitary gland. This study provides new insights into the cellular origins of iGCTs and suggests further investigation into the regulatory mechanisms that may lead to tumorigenesis.

**Supplementary Information:**

The online version contains supplementary material available at 10.1038/s41598-026-38060-2.

## Introduction

Intracranial germ cell tumors (iGCTs) are a rare type of tumor found in the central nervous system (CNS). They are histologically classified as extragonadal germ cell tumors (GCTs). According to the 2021 World Health Organization (WHO) classification of CNS tumors, GCTs include several subtypes: germinoma, mature teratoma, immature teratoma, teratoma with somatic-type malignancy, embryonal carcinoma, yolk sac tumor, choriocarcinoma, and mixed germ cell tumors^1^ Among these, germinoma makes up 60–65% of pediatric iGCTs, and the remaining subtypes are known as non-germinomatous germ cell tumors (NGGCTs)^2^.

iGCTs primarily affect children and adolescents, with a median age of diagnosis ranging from 10 to 14 years^3^. iGCTs are often found in the midline of the brain, particularly in the pineal gland and the hypothalamic-neurohypophyseal axis (HNA). A distinctive epidemiological feature is the marked sex disparity that varies by anatomical site: tumors in the pineal region show a strong male predominance (male:female ratio reported as ~ 13–15:1),^3^ whereas suprasellar/hypothalamo-neurohypophyseal tumors show a much less pronounced male predominance and may even demonstrate a slight female predominance in some cohorts (reported male:female ratios approaching ~ 1:1 or < 1 in suprasellar series)^4^. Another feature is the significant geographic differences in the incidence of iGCTs (e.g., the incidence in East Asia is 3–8 times higher than in Western countries)^5–7^. The above two features indicate the genetic background of origin of iGCTs.

GCTs typically arise in the gonads, with the testis being the most common site. However, only 3% of GCTs originate outside the gonads, with the CNS being the most frequent extragonadal site^8^. The histological similarity between GCTs in the CNS and other body parts seems to suggest a common cellular origin. However the gonadal GCTs show the opposite trend in regional prevalence (e.g., testicular GCTs have an incidence of 55/million/year in the United States but 25/million/year in Japan). So, the origin of iGCTs is still controversial. The focus of debate is whether iGCTs originate from abnormally migrating primordial germ cells (PGCs) or from transformed embryonic stem cells (ESCs). However, only a limited amount of basic research on iGCTs has been conducted due to the low incidence and difficulty obtaining tumor specimens. There is no evidence to support the presence of PGCs in the brain or extragonadal PGCs in human fetuses.

The pituitary gland has the capacity to adapt its cellular composition to meet the changing hormone demands throughout an individual’s life. Over the past 15 years, research has indicated the presence of resident stem cells within the pituitary gland^9^. Single-cell transcriptomic studies have provided insights into the developmental pathways of various pituitary cell lineages during human pituitary gland development^10^. Building on these insights, we hypothesize that the pituitary may harbor a rare population of resident cells that express germline-associated genes and share partial biological traits with primordial germ cells. We further propose that, under complex and dynamic regulation of the hypothalamic–pituitary–gonadal axis (HPGA), such cells may acquire tumorigenic phenotypes and contribute to the development of iGCTs along the hypothalamic–pituitary region.

In this study, we use the term “PGC-like” to refer to pituitary-resident cells that exhibit expression of multiple germ cell–associated markers (MVH/*DDX4*, OCT4, KIT, and PLZF) detected by histology and molecular assays, suggesting partial overlap with germline-related programs. We acknowledge that bona fide primordial germ cells are developmentally defined by embryonic origin, migratory behavior, and characteristic epigenetic reprogramming, none of which can be inferred solely from marker expression in adult tissues. Therefore, “PGC-like” is used here as a cautious descriptive term pending single-cell and functional validation.

To better understand the significance of these markers, it is important to highlight their individual roles in germ cell biology. MVH, also known as *DDX4*, is a member of the DEAD-box protein family and functions as an ATP-dependent RNA helicase with RNA-binding properties^11^. It is a well-established marker of germ cells in animals^12^. In human testicular tissues, *DDX4* expression identifies spermatogonia, spermatocytes, and spherical spermatids^13,14^. OCT3/4, also known as *POU5F1*, is a transcription factor predominantly expressed in pluripotent stem and germ cells^15^. In normal human tissues, OCT3/4 expression occurs during early embryonic development and remains highly expressed in embryonic stem cells and germ cells^16^. A systematic review and meta-analysis have demonstrated that OCT3/4 is a highly reliable immunohistochemical marker for diagnosing iGCTs^17^. C-kit serves as the receptor for the stem cell factor (SCF), also referred to as kit ligand (KITL). Its expression spans various cell types, including germ cells, hematopoietic stem cells, hematopoietic progenitor cells, and melanocytes^18^ The KITL-KIT signaling pathway plays critical roles in gametogenesis, fertility, hematopoiesis, and melanogenesis^19^ PLZF, also known as *ZBTB16*, is crucial for the self-renewal of spermatogonial stem cells (SSCs). In mice, PLZF is co-expressed with OCT4 in undifferentiated spermatogonia^20^ Although the exact function of PLZF in SSCs remains unclear, recent studies have revealed that PLZF acts as an epigenetic regulator, contributing to the maintenance and self-renewal of germ cells^21^.

In this study, we compared germ cell marker expression in human normal pituitary tissues, pituitary neuroendocrine tumors (PitNETs), and pituitary-region germ cell tumors using immunohistochemistry, western blotting, and qRT-PCR, with mouse testes as positive control tissue and Jurkat cells as a non-germline human reference sample. Here, we detected expression of MVH/DDX4, OCT4, KIT, and PLZF in normal human pituitary tissues, with higher levels in pituitary tumors and the highest expression in pituitary germ cell tumors, supporting the presence of germline-marker–positive cells within the pituitary.

## Materials and methods

###  Human and mouse samples

The study subjects were patients from the Neurosurgery Department of Peking Union Medical College Hospital who underwent pituitary surgery, with postoperative samples collected from their pituitary glands, pituitary tumors, and intracranial germ cell tumors. This study was approved by the Peking Union Medical College Hospital Ethics Committee, Chinese Academy of Medical Sciences, Beijing, China. All participants signed informed consent forms prior to the experiment, fully understanding the research objectives, methods, and potential risks. The research strictly adhered to the ethical principles of the Declaration of Helsinki, ensuring the privacy and information security of participants.Clinicopathological variables were extracted from medical records when available and are summarized in Supplementary Table S1, including specimen category, diagnosis/subtype, anatomical location, and key immunohistochemical profile.

Ten male C57BL/6J mice (7–8 weeks old, 20 ± 2 g) were provided by Sipeifu (Beijing) Biotechnology Co., Ltd. The animals were maintained under controlled environmental conditions with a 12-h light/dark cycle and free access to food and water. All experimental procedures were approved by the Institutional Animal Care and Use Committee of the Institute of Basic Medical Sciences, Beijing, China . All methods were performed in accordance with the relevant guidelines and regulations, and this study is reported in accordance with the ARRIVE guidelines.

After one week of acclimatization, the mice were deeply anesthetized with isoflurane inhalation (2–3% in oxygen) and euthanized by CO₂ inhalation at a gradual fill rate of approximately 20–30% of the chamber volume per minute, followed by cervical dislocation, in accordance with the AVMA Guidelines for the Euthanasia of Animals (2020). Mouse testes were used as a positive control tissue for germ cell marker expression in western blotting and/or immunostaining assays.

### Cell culture

Jurkat cells (human acute T lymphoblastic leukemia cell line; suspension growth) were obtained from the Cell Resource Center, Institute of Basic Medical Sciences, Chinese Academy of Medical Sciences (CAMS), Beijing, China (Resource ID: 1101HUM-PUMC000347). Cells were cultured in RPMI-1640 supplemented with 15% fetal bovine serum (FBS) and 1% penicillin/streptomycin at 37 °C in a humidified incubator with 5% CO₂, and harvested at the logarithmic growth phase for RNA and protein extraction. Mycoplasma testing provided by the supplier was negative (culture-based assay), and cell identity was supported by the supplier’s STR profile. Jurkat cells are an established human cell line obtained from an institutional cell resource center; therefore, no additional ethics approval was required for their use in this study. All procedures complied with institutional biosafety regulations. Jurkat cells were used as a human non-germline reference sample for relative expression comparisons and were not intended to model pituitary biology.

### Chemicals and reagents

Xylene and formaldehyde solutions were purchased from Sinopharm Chemical Reagent Co., Ltd. (Shanghai, China), while hematoxylin, horseradish peroxidase (HRP)-conjugated secondary antibodies, and DAB chromogenic kits were obtained from Beijing Zhong Shan—Golden Bridge Biological Technology Co., Ltd. (Beijing, China). Information on other antibodies used in the study is detailed in Table 1.

**Table 1:** Antibodies used in the study.

Antibody	Vender	Catalog number	RRID	Host species	Working dilution	Use
MVH	Abcam	Ab13840	AB_443012	Rabbit	1:800	IHC/WB
PLZF	Santa Cruz Biotechnology	sc-28319	AB_2218941	Mouse	1:200	IHC/WB
C-kit	Abcam	ab256345	AB_2891166	Rabbit	1:800	IHC/WB
OCT4	Proteintech	60,242-1-lg	AB_2881364	Mouse	1:400	IHC/WB

### Immunohistochemical staining

Human normal pituitary tissues, pituitary tumor tissues (PitNETs; subtype information provided in Table S1, and pituitary germ cell tumor tissues were fixed in 10% neutral-buffered formalin, paraffin-embedded, and sectioned at 4 μm thickness. Sections were deparaffinized in xylene and rehydrated through graded ethanol. Antigen retrieval was performed in sodium citrate buffer (pH 6.0) using heat-mediated retrieval. Endogenous peroxidase activity was blocked with 3% hydrogen peroxide for 20 min, followed by blocking with goat serum for 1 h at room temperature. Sections were incubated with primary antibodies (Table 1) overnight at 4 °C, washed with PBS, and incubated with HRP-conjugated secondary antibodies for 1 h at room temperature. Signals were visualized using DAB and counterstained with hematoxylin. Negative controls were prepared by omitting the primary antibody. Images were acquired at 40 ×magnification; scale bars are indicated in the figure panels and legends. Primary antibodies used for IHC (including anti-DDX4/MVH, anti-OCT4, anti-KIT, and anti-PLZF) and their sources/dilutions are listed in Table 1.

### Western blotting

Mouse testes and human tissues were homogenized in RIPA buffer supplemented with protease inhibitors on ice and clarified by centrifugation. Protein concentration was determined using the bicinchoninic acid (BCA) assay^22^. Equal amounts of protein (20 μg per lane) were separated by SDS–PAGE (10% gels) and transferred to PVDF membranes by electrophoretic transfer^23^. Membranes were blocked with 5% non-fat milk in TBST for 1 h at room temperature, incubated with primary antibodies (Table 1) overnight at 4 °C, and then with HRP-conjugated secondary antibodies for 1 h at room temperature. Bands were visualized using a Bio-Rad chemiluminescence imager. Densitometric quantification was performed in ImageJ, and target band intensities were normalized to β-actin. Antibodies used for western blotting and their working dilutions are provided in Table 1.

### Real-time quantitative reverse transcription PCR (qRT-PCR)

Total RNA was extracted from fresh/frozen tissues using TRIzol according to the manufacturer’s protocol. RNA concentration and purity were assessed (A260/280). Equal amounts of RNA were reverse-transcribed into cDNA using a reverse transcription kit. qPCR was performed using SYBR Green chemistry on a real-time PCR system with gene-specific primers (Table 2). Each sample was run in technical duplicates/triplicates. Relative expression was calculated using the **2^-ΔΔCt** method with β-actin as the internal control and the specified calibrator group. Primer sequences for MVH/*DDX4*, OCT4/*POU5F1*, *KIT*, PLZF/*ZBTB16*, and β-actin are listed in Table 2.

**Table 2:** Primers used for real-time qRT-PCR in humans.

Genes	Forward primers (5′- > 3′)	Reverse primers(5′- > 3′)
MVH	CAGCAGGTTTGAAGATGGTGA	GGTCTAAGTCATTATCGCCTCTC
C-kit	GCACAATGGCACGGTTGAAT	GTGGGGATGGATTTGCTCTTTG
PLZF	TCGGCTCTCGGCGGG	CAATAGAGGAGGCGAGAGCG
OCT4	GCTGGATGTCAGGGCTCTTT	AACCACACTCGGACCACATC
β-actin	ACAGAGCCTCGCCTTTGC	GATATCATCATCCATGGTGAGCTGG

### Data analysis and statistics

Data analysis and statistics. For immunohistochemistry, staining was quantified using an H-score (intensity × percentage). Scoring was performed by two independent observers blinded to group assignment. For western blotting, band intensities were quantified using ImageJ and normalized to β-actin. For qRT-PCR, Ct values from technical duplicates/triplicates were analyzed using the 2^^-ΔΔCt^ method. Data are presented as mean ± SD. Normality was assessed using the Shapiro–Wilk test. For comparisons among multiple groups, one-way ANOVA with Tukey’s post hoc test was used for normally distributed data; otherwise, Kruskal–Wallis with Dunn’s correction was applied. Statistical analyses were conducted using GraphPad Prism 9.0, with p < 0.05 considered significant.

## Results

**Fig. 1:**
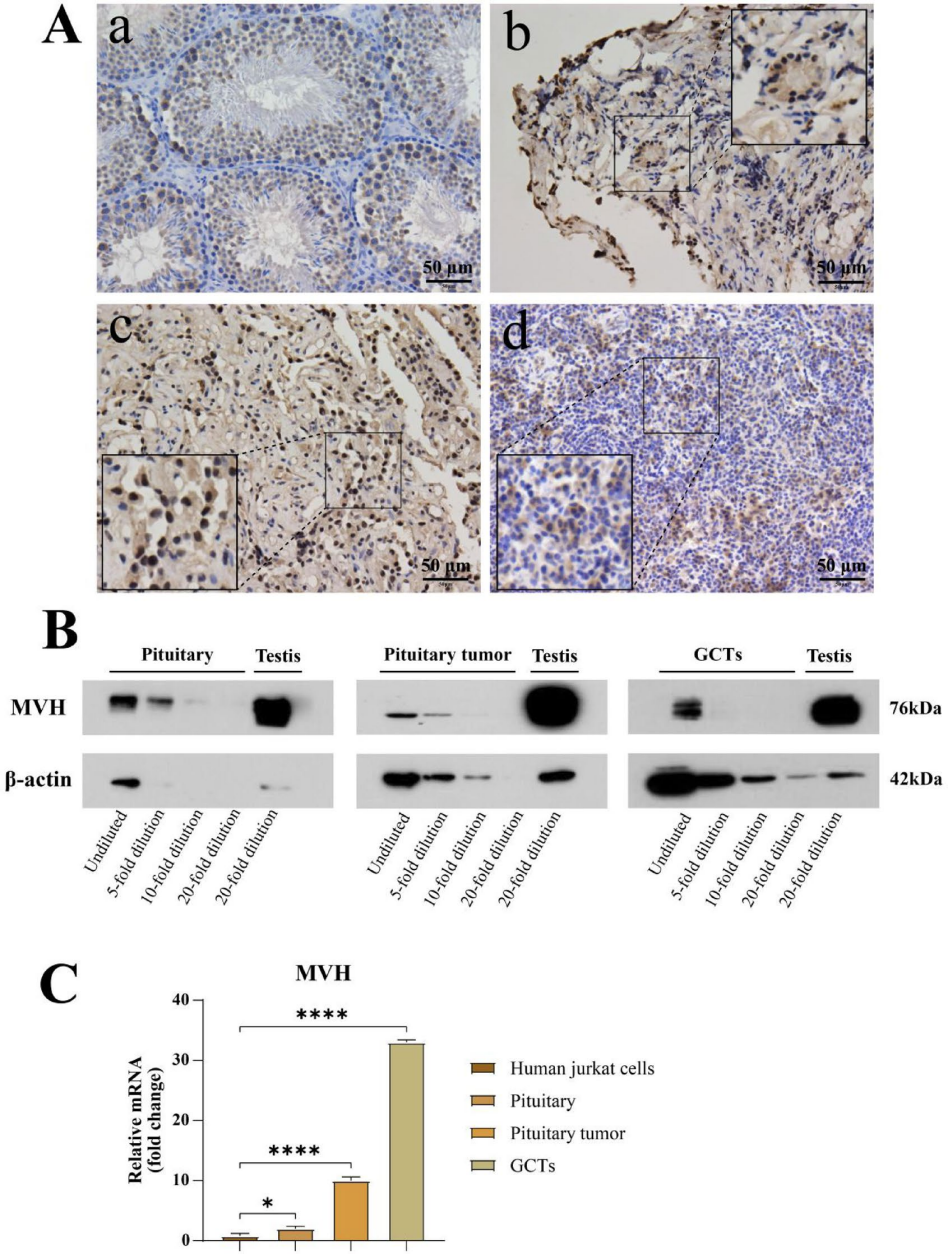
Expression of the germ cell marker MVH/DDX4 in the pituitary and pituitary-related tumors. (**A**) Representative immunohistochemistry (IHC) for MVH in (a) mouse testes, (b) human pituitary, (c) PitNET and (d) pituitary-region iGCT. IHC was semi-quantified by H-score from three non-overlapping regions of interest (ROIs) per panel (n_ROI = 3) using 0–3 + intensity categories. Mean ± SD H-scores: testes 154.22 ± 21.40; pituitary 105.27 ± 40.79; PitNET 131.08 ± 48.26; iGCT 117.00 ± 17.51. (**B**) Immunoblot analysis of MVH in indicated tissues. Blots were cropped for clarity; uncropped blots are provided in Supplementary Figure. (C) MVH gene expression in human Jurkat cells, human pituitary, pituitary tumors, and germ cell tumors (*P < 0.05, **P < 0.01, ***P < 0.001, ****P < 0.0001).

MVH/DDX4 protein is primarily expressed in spermatogonial cells, various stages of spermatocytes, and round spermatids in the testes of mice, where it is involved in regulating the development of germ cells and maintaining their stemness^24^. Immunohistochemical staining of human normal pituitary, pituitary tumors, and pituitary germ cell tumors also shows expression of the MVH protein, with positive signals primarily localized in the cytoplasm and nucleus. H-score quantification is provided in the figure legends (Fig. 1).Western blot (WB) results demonstrate high expression of MVH protein in mouse testes, as well as varying levels of expression in human normal pituitary, pituitary tumors, and pituitary germ cell tumors, with the most significant expression observed in pituitary germ cell tumors. PCR analysis indicates that, compared to the human Jurkat cell control, MVH gene expression in human normal pituitary, pituitary tumors, and pituitary germ cell tumors is 2, 10, and 33 times that of the control, respectively (Fig. 1).Notably, MVH/*DDX4* protein and mRNA levels did not show a strict linear correlation across specimen categories. Such discordance may reflect post-transcriptional regulation, differential protein stability, variability in tissue cellular composition, and methodological differences between bulk transcript and protein measurements.

**Fig. 2:**
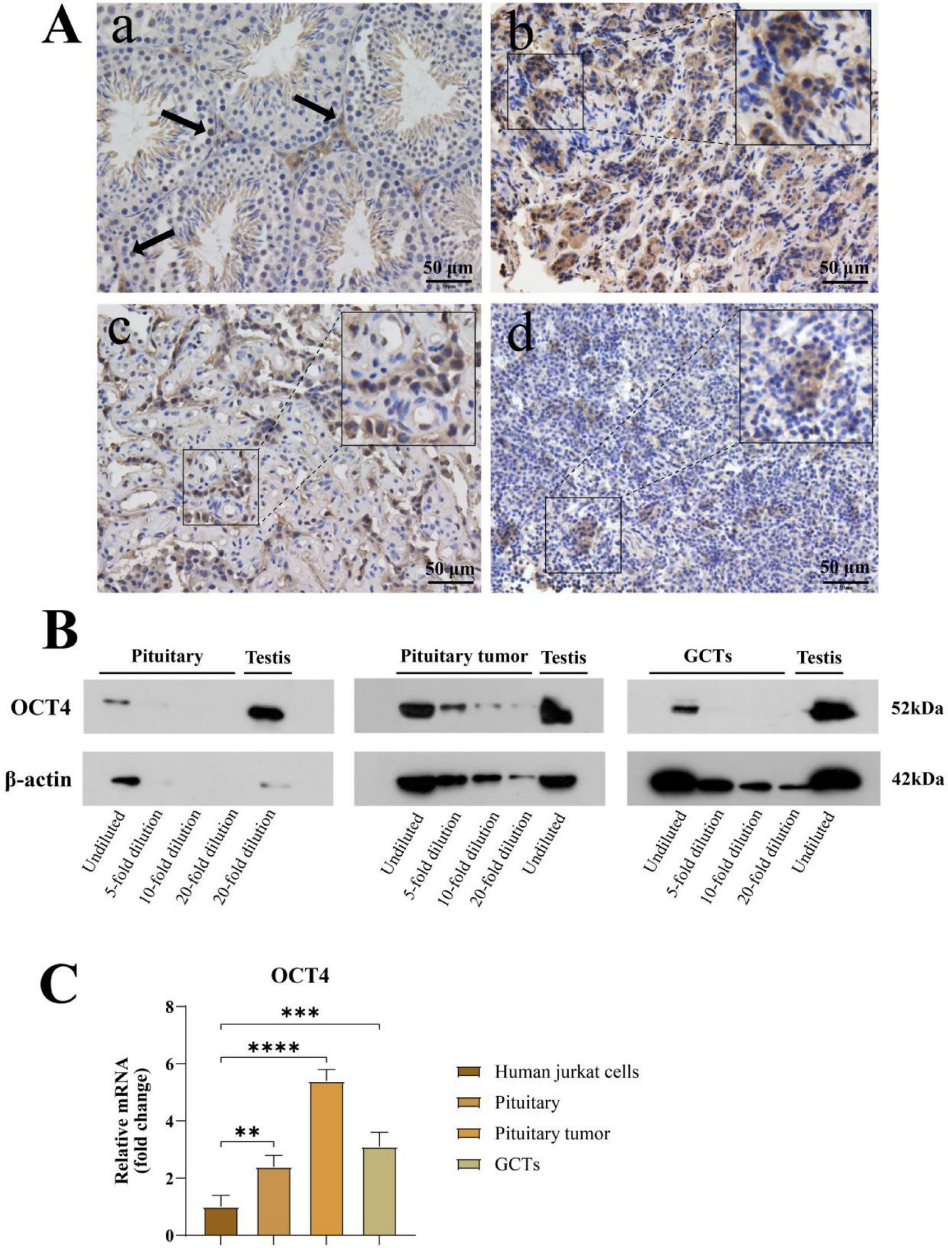
Expression of the germ cell marker OCT4 in the pituitary and pituitary-related tumors. (**A**) Representative IHC for OCT4/POU5F1 in (a) mouse testes, (b) human pituitary, (c) PitNET and (d) pituitary-region iGCT. In these sections, OCT4/POU5F1 immunoreactivity is predominantly cytoplasmic/perinuclear. H-scores were calculated from three non-overlapping ROIs per p $\mathrm{anel}({\mathrm{n}}_{\mathrm{R}}\mathrm{OI}=3)\mathrm{usin}$ g 0–3 + intensity categories. Mean ± SD H-scores: testes 112.47 ± 17.03; pituitary 112.64 ± 4.77; PitNET 149.88 ± 1.69; iGCT 98.08 ± 11.01. (**B**) Immunoblot analysis of OCT4/POU5F1 in indicated tissues. Blots were cropped for clarity; uncropped blots are provided in Supplementary Figure. (C) OCT4 gene expression in human Jurkat cells, human pituitary, pituitary tumors, and germ cell tumors (*P < 0.05, **P < 0.01, ***P < 0.001, ****P < 0.0001).

In mouse testes, OCT4 protein is primarily expressed in spermatogonial cells and early spermatocytes, playing a crucial role in maintaining the stemness and proliferative capacity of these cells^25^. In human normal pituitary, pituitary tumors, and pituitary germ cell tumors, OCT4 immunoreactivity was predominantly cytoplasmic with a perinuclear accentuation, whereas convincing nuclear positivity was limited and only observed in a small subset of cells.H-score quantification is provided in the figure legends (Fig. 2). WB results further confirm the expression of OCT4 protein in human normal pituitary, pituitary tumors, and pituitary germ cell tumors. Protein samples were serially diluted, and the results showed a gradient decrease in OCT4 protein expression. PCR analysis indicates that, compared to the human Jurkat cell control, OCT4 gene expression in pituitary tumors is 5 times that of the control, while in normal pituitary and pituitary germ cell tumors, the expression is 2 and 3 times that of the control, respectively (Fig. 2).

**Fig. 3:**
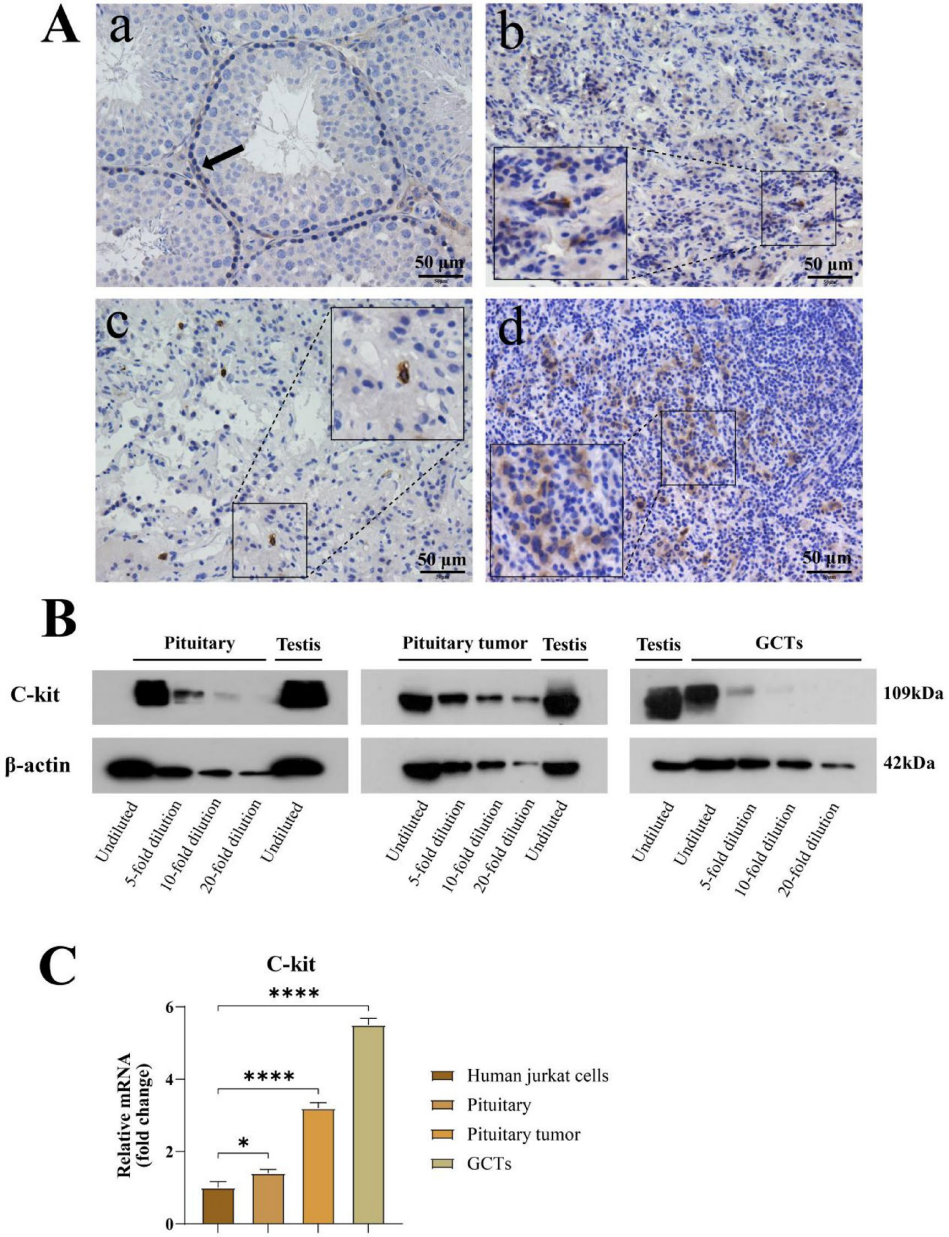
Expression of the germ cell marker C-kit in the pituitary and pituitary-related tumors.(**A**) Representative IHC for KIT/C-kit (CD117) in (a) mouse testes, (b) human pituitary, (c) PitNET and (d) pituitary-region iGCT. H-scores were calculated from three non-overlapping ROIs per panel (n_ROI = 3) using 0–3 + intensity categories. Mean ± SD H-scores: testes 141.30 ± 23.22; pituitary 97.41 ± 5.28; PitNET 56.63 ± 38.15; iGCT 58.84 ± 48.27. (**B**) Immunoblot analysis of KIT/C-kit in indicated tissues. Blots were cropped for clarity; uncropped blots are provided in Supplementary Figure.(C) C-kit gene expression in human Jurkat cells, human pituitary, pituitary tumors, and germ cell tumors (*P < 0.05, **P < 0.01, ***P < 0.001, ****P < 0.0001).

KIT protein is a receptor tyrosine kinase that is primarily expressed in spermatogonial cells and germ stem cells^26^. It also shows positive expression in human normal pituitary, pituitary tumors, and pituitary germ cell tumors, with the positive cells typically exhibiting a large nucleus and a round shape.H-score quantification is provided in the figure legends (Fig. 3). WB results further confirm the expression of C-kit protein in human pituitary tissue. PCR analysis shows that the expression of the C-kit gene in human normal pituitary, pituitary tumors, and pituitary germ cell tumors is 1.4, 3, and 5.5 times that of the human Jurkat cell control, respectively (Fig. 3).

**Fig. 4:**
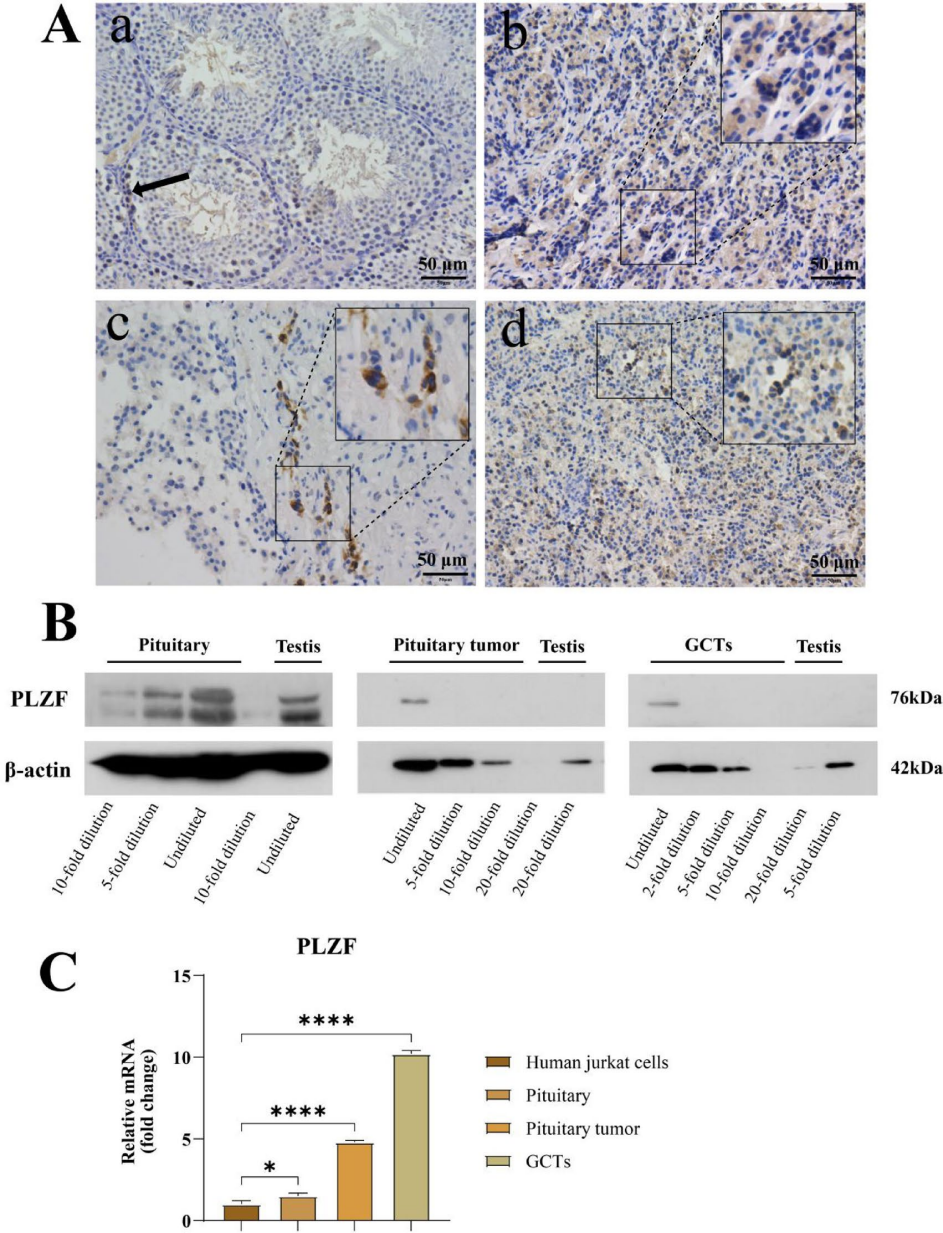
Expression of the Germ Cell Marker PLZF in the Pituitary and Pituitary-Related Tumors. (**A**) Representative IHC for PLZF/ZBTB16 in (a) mouse testes, (b) human pituitary, (c) PitNET and (d) pituitary-region iGCT. H-scores were calculated from three non-overlapping ROIs per panel (n_ROI = 3) using 0–3 + intensity categories. Mean ± SD H-scores: testes 100.37 ± 23.08; pituitary 87.71 ± 13.76; PitNET 137.18 ± 42.48; iGCT 96.91 ± 21.21. (**B**) Immunoblot analysis of PLZF/ZBTB16 in indicated tissues. Blots were cropped for clarity; uncropped blots are provided in Supplementary Figure. (C) PLZF gene expression in human Jurkat cells, human pituitary, pituitary tumors, and germ cell tumors (*P < 0.05, **P < 0.01, ***P < 0.001, ****P < 0.0001).

PLZF protein is primarily expressed in spermatogonial cells and plays a crucial role in maintaining their self-renewal, proliferation, and undifferentiated state^27^. In human normal pituitary, PLZF protein is predominantly expressed in the cytoplasm in a clustered pattern, while in pituitary tumors and pituitary germ cell tumors, its positive expression is mainly confined to the nucleus.H-score quantification is provided in the figure legends (Fig. 4). WB results show that PLZF protein expression is relatively low in pituitary tumors, with varying levels of expression in other tissues. PCR analysis indicates that the expression of the PLZF gene in pituitary tumors is 1.6 times that of the human Jurkat cell control, while in normal pituitary and pituitary germ cell tumors, PLZF gene expression is 5 and 10 times that of the control, respectively (Fig. 4).

Across MVH/DDX4, OCT4, KIT, and PLZF, we observed consistent marker signals in normal pituitary tissues with increased expression in PitNETs and the highest expression in pituitary germ cell tumors. While these findings support a germline-associated molecular signature in pituitary tissues, marker expression alone is insufficient to definitively assign cellular identity. Future studies should evaluate co-expression patterns within the same cells, subcellular localization, and cell-type specificity using multiplexed single-cell and spatial assays.

## Discussion

In this study, we employed immunohistochemical staining, Western blotting, and qRT-PCR to examine the expression levels of four germ cell markers- MVH, OCT4, C-kit, and PLZF -in normal human pituitary glands compared to human Jurkat cells. We found that the expression levels of these markers were higher in the normal pituitary gland. Furthermore, in both pituitary tumors and pituitary GCTs, the expression levels of these markers were significantly increased to various extents. Across immunohistochemistry, western blotting, and qRT-PCR, these convergent data support the presence of a germline-like molecular signature within pituitary tissue. Importantly, marker expression alone cannot define lineage; our findings therefore motivate a testable hypothesis rather than establishing a definitive cell of origin.

The origins of iGCTs have been debated for decades. Nearly half a century ago, Teilum proposed a theory regarding the origin of extragonadal GCTs. He suggested that these tumors arise from ectopic PGCs that migrate abnormally during the early stages of embryonic development. These misplaced PGCs are thought to undergo malignant transformation as the embryo develops in the brain or other midline regions of the body^28^. Subsequently, based on the DNA methylation profiles of human iGCTs and mouse PGCs^29^. Fukushima et al. proposed that germinomas originate from migrating PGCs. According to the Sano theory, ESCs from the embryonic disc and/or extraembryonic tissues migrate during the formation of the primitive streak, enter the primitive groove, and then migrate to the neural plate. After the neural tube forms, these ESCs become encapsulated within the neural axis and eventually develop into GCTs^30^. However, whether originating from PGCs or ESCs, the mechanisms by which they preferentially migrate to the future diencephalon region, remain dormant without undergoing apoptosis, and later transform into iGCTs remain unclear. Additionally, these theories do not fully explain the observed preferences of iGCTs for specific patient ages, genders, and races.

Neural stem cells (NSCs) have also been considered as a potential source. Tan and Scotting suggested that teratomas may arise from NSCs, independent of the germ cell lineage, while other GCTs could be directly linked to teratomas and share a similar non-germ cell origin. This implies that the transformation of neural stem cells might underlie the development of all iGCTs^31^. OCT4, a crucial gene for the pluripotency of ESCs, can activate NSCs to form teratomas^32^. Human induced pluripotent stem cells (iPSCs) derived from somatic and neural stem cells have been shown to form teratomas upon transplantation into mice and to give rise to various types of GCTs, including embryonal carcinomas, immature teratomas (often containing primitive neuroectodermal tissue), yolk sac tumors, and somatic-type malignant tumors. However, germinomas have never been observed in iPSC-derived tumors^33^, which does not explain the predominance of germinomas among iGCTs. Moreover, if NSCs are indeed the progenitors of iGCTs, it remains unclear why the incidence of iGCTs is higher in the midline region rather than in the lateral ventricles, where NSCs are most abundant.

Current models for iGCTs ontogeny—including aberrant migration of primordial germ cells, transformation of embryonic stem-like cells, and potential contributions from neural stem cells—capture aspects of early development but leave key clinicopathological features insufficiently explained, notably the strong enrichment along the hypothalamic–pituitary axis and the long latency before clinical presentation. A pituitary-resident, germline-marker–positive compartment offers a complementary framework that aligns anatomically with the sellar/suprasellar distribution of many tumors and provides a local niche in which dormant precursor-like cells could persist. This does not negate developmental hypotheses; rather, it introduces an additional, anatomically grounded candidate substrate that can be directly interrogated in postnatal tissue.

A key question raised by our data is the identity of germline-marker–positive cells within the pituitary and their relationship to established pituitary stem/progenitor compartments. Prior work in rodents demonstrates that the adult pituitary contains a structured stem/progenitor niche in the marginal zone (MZ), as well as scattered stem/progenitor marker–positive cells and small clusters within the adenoparenchyma (AP), and that these compartments fluctuate with physiological reproductive states. In particular, stem/progenitor markers (including *GFRA2*,* SOX2*, and *SOX9*) have been detected in the MZ and AP, with marker abundance and proliferative activity varying across gestation and lactancy, consistent with endocrine-driven remodeling of pituitary cellular composition^34^. These observations are relevant to our findings because they establish that the adult pituitary harbors rare, plastic, non-endocrine compartments whose molecular state can shift with hormonal demand. Notably, this physiological literature also reports OCT4 expression within pituitary stem/progenitor compartments and describes context-dependent subcellular redistribution, including conditions where OCT4 signal is higher in the cytoplasm than in the nucleus^34^. This provides a pituitary-centered rationale for interpreting OCT4 immunoreactivity cautiously in adult tissue and reinforces the need for orthogonal localization validation.

Evidence from pathological modeling further indicates that pituitary stem/progenitor niches are remodeled during tumorigenic progression. In an estradiol benzoate–driven hyperplastic/adenomatous model, stem/tumor stem cell–associated markers (including GFRA2, Nestin, and *CD133*) increase early within the MZ niche, whereas later stages show increased representation of markers such as *GFRA2* and *SOX9* within the AP niche, suggesting dynamic niche redistribution during tumor development^35^. The same work highlights tissue-level heterogeneity and partial co-expression among proposed stem/progenitor markers (e.g.,* SOX2/GFRa2*, Nestin/*CD133*, *SOX9*/*CD133*), consistent with a state-based or hierarchical organization rather than a single uniform population^35^. Together, these findings emphasize that pituitary stem/progenitor programs are plastic, spatially organized, and responsive to endocrine perturbation in ways that may be relevant to neoplastic susceptibility. They also underscore an interpretive limitation of our own bulk assays: germline-marker signals could reflect a rare resident lineage, a transient cellular state within stem/progenitor compartments, or another pituitary cell population with partial marker overlap.

The endocrine context provides a plausible mechanistic entry point for future testing. We hypothesize that dynamic regulation of the HPGA may influence the survival, proliferation, or fate stability of germline-marker–positive cells, thereby shaping susceptibility to transformation; however, this remains untested in the present study. The physiological and pathological stem/progenitor literature cited above supports the broader concept that changing hormonal milieus can remodel pituitary niches and marker programs^34,35^. A rigorous path forward is therefore to relate germline-marker expression and cellular state to endocrine variables across life stages, define hormone receptor expression and downstream signaling at single-cell resolution, and evaluate causality through controlled endocrine perturbation in vitro and in vivo. Such studies would also clarify whether endocrine dynamics contribute to the age- and sex-associated patterns observed in iGCT epidemiology.

The detection of germ cell–associated markers in normal pituitary tissue also underscores that marker positivity is not synonymous with tumorigenesis. iGCTs remain rare, implying that additional events are required for malignant transformation^36^. A parsimonious explanation is a multi-hit process in which genetic susceptibility, epigenetic dysregulation, and permissive microenvironmental cues—including endocrine signaling—cooperate to promote lineage instability and unchecked growth^37^. Under this view, germline-marker–positive cells represent a potential cellular substrate, while progression depends on the convergence of multiple enabling factors. This framework can be tested in larger, clinically annotated cohorts integrating genomic and epigenomic profiling with single-cell and spatial mapping^38^.

Our findings have direct clinical relevance for sellar/suprasellar lesions. A pituitary-resident, germline-marker–positive niche provides a biologically grounded framework to sharpen differential diagnosis in atypical cases where imaging, endocrine phenotypes, and routine histology do not confidently distinguish PitNETs from pituitary-region iGCTs. In this context, our data support more systematic use of germ cell–associated marker panels (including OCT4*/POU5F1* and KIT/*CD117*) to improve classification and potentially reduce diagnostic delay. The same framework also enables testable, mechanism-informed translational hypotheses: KIT-linked receptor tyrosine kinase signaling, OCT4-associated stemness/pluripotency networks, and endocrine or microenvironmental cues may cooperate to promote lineage instability and tumorigenesis. These candidates are well suited to validation by pathway-level profiling and functional perturbation, particularly with single-cell and spatially resolved approaches.

Several limitations temper the interpretation. We have not isolated the putative germline-marker–positive cells, demonstrated multi-marker co-localization at single-cell resolution, or established functional properties; thus, cellular identity remains inferential. In addition, our IHC semi-quantification was performed at the ROI level from representative sections and therefore does not constitute independent biological replication, limiting the strength of between-group inference; larger, clinically annotated cohorts with specimen-level quantification and appropriate hierarchical modelling will be required to robustly test group differences. More broadly, marker expression is not cell-type exclusive and may reflect cellular heterogeneity within bulk tissue measurements. Addressing these limitations will require multiplexed co-localization strategies (e.g., multiplex immunofluorescence or RNA in situ hybridization), single-cell RNA sequencing and spatial transcriptomics to define identity and niche architecture, and functional perturbation—including CRISPR-based approaches—to test causality. Together, these efforts will be essential to determine whether pituitary-resident germline-marker–positive cells contribute to iGCT initiation and to delineate the molecular circuitry linking endocrine regulation to tumorigenesis.

## Conclusion

The findings of this study suggest that PGC-like cells exist within the normal human pituitary gland, as evidenced by the expression of key germ cell markers such as MVH, OCT4, C-kit, and PLZF. These markers were found to be upregulated in pituitary tumors and pituitary germ cell tumors. The complex regulation of the HPGA could influence the differentiation of these PGC-like cells, contributing to the formation of iGCTs. This novel insight into the cellular origin of iGCTs challenges existing theories. Further research is needed to isolate and fully characterize these PGC-like cells, explore the molecular mechanisms driving tumorigenesis, and investigate how these findings could translate into more accurate diagnostic, prognostic, and therapeutic strategies for iGCTs.

## Supplementary Information

Below is the link to the electronic supplementary material.

Supplementary Material 1

Supplementary Material 2

## Data Availability

All data supporting the findings of this study are available within the article and its Supplementary Information. Additional raw data are available from the corresponding author upon reasonable request.

## References

[1] D.N. Louis, A. Perry, P. Wesseling, D.J. Brat, I.A. Cree, D. Figarella-Branger, C. Hawkins, H.K. Ng, S.M. Pfister, G. Reifenberger, R. Soffietti, A. von Deimling, D.W. Ellison, The 2021 WHO Classification of Tumors of the Central Nervous System: a summary, Neuro-oncology, 23 (2021) 1231–1251. 10.1093/neuonc/noab106 PMC8328013 34185076

[2] Jennings, M. T., Gelman, R. & Hochberg, F. Intracranial germ-cell tumors: Natural history and pathogenesis. *J Neurosurg* **63**, 155–167 (1985). 2991485 10.3171/jns.1985.63.2.0155

[3] H. Gittleman, G. Cioffi, T. Vecchione-Koval, Q.T. Ostrom, C. Kruchko, D.S. Osorio, J.L. Finlay, J.S. Barnholtz-Sloan, Descriptive epidemiology of germ cell tumors of the central nervous system diagnosed in the United States from 2006 to 2015, Journal of neuro-oncology, 143 (2019) 251-260. 10.1007/s11060-019-03173-4 31025275

[4] Zhang, Y. et al. Delays in diagnosis of pediatric histologically confirmed sellar germ cell tumors in China: A retrospective risk factor analysis. *World Neurosurg.* **122**, e472–e479 (2019). 30366141 10.1016/j.wneu.2018.10.082

[5] Jung, K. W., Ha, J., Lee, S. H., Won, Y. J. & Yoo, H. An updated nationwide epidemiology of primary brain tumors in republic of Korea. *Brain Tumor Res. Treat.* **1**, 16–23 (2013). 24904884 10.14791/btrt.2013.1.1.16 PMC4027117

[6] Murray, M. J. et al. Consensus on the management of intracranial germ-cell tumours, The Lancet. *Oncology* **16**, e470–e477 (2015). 26370356 10.1016/S1470-2045(15)00244-2

[7] Murray, M. J., Horan, G., Lowis, S. & Nicholson, J. C. Highlights from the third international central nervous system germ cell tumour symposium: Laying the foundations for future consensus. *Ecancermedicalscience* **7**, 333 (2013). 23861728 10.3332/ecancer.2013.333 PMC3709531

[8] Arora, R. S., Alston, R. D., Eden, T. O., Geraci, M. & Birch, J. M. Comparative incidence patterns and trends of gonadal and extragonadal germ cell tumors in England, 1979 to 2003. *Cancer* **118**, 4290–4297 (2012). 22252431 10.1002/cncr.27403

[9] Laporte, E., Vennekens, A. & Vankelecom, H. Pituitary remodeling throughout life: Are resident stem cells involved?. *Front. Endocrinol.* **11**, 604519 (2020). 10.3389/fendo.2020.604519 PMC7879485 33584539

[10] Zhang, S. et al. Single-cell transcriptomics identifies divergent developmental lineage trajectories during human pituitary development. *Nat. Commun.* **11**, 5275 (2020). 33077725 10.1038/s41467-020-19012-4 PMC7572359

[11] Amirian, M., Azizi, H., Hashemi Karoii, D. & Skutella, T. VASA protein and gene expression analysis of human non-obstructive azoospermia and normal by immunohistochemistry, immunocytochemistry, and bioinformatics analysis. *Sci. Rep.* **12**, 17259 (2022). 36241908 10.1038/s41598-022-22137-9 PMC9568577

[12] Adashev, V. E., Kotov, A. A. & Olenina, L. V. RNA helicase vasa as a multifunctional conservative regulator of gametogenesis in eukaryotes. *Curr. Issues Mol. Biol.* **45**, 5677–5705 (2023). 37504274 10.3390/cimb45070358 PMC10378496

[13] Adashev, V. E. et al. Stellate genes and the piRNA pathway in speciation and reproductive isolation of drosophila melanogaster. *Front. Genet.* **11**, 610665 (2020). 33584811 10.3389/fgene.2020.610665 PMC7874207

[14] W. Li, P. Zhang, X. Wu, X. Zhu, H. Xu, A Novel Dynamic Expression of vasa in Male Germ Cells during Spermatogenesis in the Chinese Soft-Shell Turtle (Pelidiscus sinensis), Journal of experimental zoology. Part B, Molecular and developmental evolution, 328 (2017) 230-239. 10.1002/jez.b.22728 28191733

[15] Li, X. et al. Enrichment of Oct3/4-positive cells from a human bronchial epithelial cell line. *APMIS : Acta Pathologica, Microbiologica, Et Immunologica Scandinavica* **121**, 612–625 (2013). 23216104 10.1111/apm.12028

[16] Ngan, K. W. et al. Immunohistochemical expression of OCT4 in primary central nervous system germ cell tumours. *J. Clin. Neurosci. Off. J. Neurosurg. Soc. Australasia* **15**, 149–152 (2008). 10.1016/j.jocn.2006.08.013 17997317

[17] Zhang, Y. et al. OCT3/4 is a potential immunohistochemical biomarker for diagnosis and prognosis of primary intracranial germ cell tumors: A systematic review and meta-analysis. *Front. Neurosci.* **17**, 1169179 (2023). 37476834 10.3389/fnins.2023.1169179 PMC10354551

[18] Luan, Y., So, W., Dong, R., Abazarikia, A. & Kim, S. Y. KIT in oocytes: A key factor for oocyte survival and reproductive lifespan. *EBioMedicine* **106**, 105263 (2024). 39067135 10.1016/j.ebiom.2024.105263 PMC11338130

[19] Lennartsson, J. & Rönnstrand, L. Stem cell factor receptor/c-Kit: from basic science to clinical implications. *Physiol. Rev.* **92**, 1619–1649 (2012). 23073628 10.1152/physrev.00046.2011

[20] Buaas, F. W. et al. Plzf is required in adult male germ cells for stem cell self-renewal. *Nat. Genet.* **36**, 647–652 (2004). 15156142 10.1038/ng1366

[21] Du, X. et al. PLZF protein forms a complex with protein TET1 to target TCF7L2 in undifferentiated spermatogonia. *Theriogenology* **215**, 321–333 (2024). 38128225 10.1016/j.theriogenology.2023.12.015

[22] Smith, P. K. et al. Measurement of protein using bicinchoninic acid. *Anal. Biochem.* **150**, 76–85 (1985). 3843705 10.1016/0003-2697(85)90442-7

[23] Towbin, H., Staehelin, T. & Gordon, J. Electrophoretic transfer of proteins from polyacrylamide gels to nitrocellulose sheets: procedure and some applications. *Proc. Natl. Acad. Sci. U.S.A.* **76**, 4350–4354 (1979). 388439 10.1073/pnas.76.9.4350 PMC411572

[24] D.H. Castrillon, B.J. Quade, T.Y. Wang, C. Quigley, C.P. Crum, 2000 The human <i>VASA</i> gene is specifically expressed in the germ cell lineage, 97 9585–9590. 10.1073/pnas.160274797 PMC16908 10920202

[25] T. Garcia, M.C. Hofmann, Isolation of undifferentiated and early differentiating type A spermatogonia from Pou5f1-GFP reporter mice, Methods in molecular biology (Clifton, N.J.), 825 (2012) 31–44. 10.1007/978-1-61779-436-0_3 PMC3314309 22144234

[26] Busada, J. T. et al. Retinoic acid regulates Kit translation during spermatogonial differentiation in the mouse. *Dev. Biol.* **397**, 140–149 (2015). 25446031 10.1016/j.ydbio.2014.10.020 PMC4268412

[27] M. Sharma, A. Srivastava, H.E. Fairfield, D. Bergstrom, W.F. Flynn, R.E. Braun, Identification of EOMES-expressing spermatogonial stem cells and their regulation by PLZF, Elife, 8 (2019). 10.7554/eLife.43352 PMC6544432 31149899

[28] Teilum, G. Classification of endodermal sinus tumour (mesoblatoma vitellinum) and so-called “embryonal carcinoma” of the ovary. *Acta Pathol. Microbiol. Scand.* **64**, 407–429 (1965). 5318716 10.1111/apm.1965.64.4.407

[29] Fukushima, S. et al. Genome-wide methylation profiles in primary intracranial germ cell tumors indicate a primordial germ cell origin for germinomas. *Acta Neuropathol.* **133**, 445–462 (2017). 28078450 10.1007/s00401-017-1673-2

[30] Sano, K. Pathogenesis of intracranial germ cell tumors reconsidered. *J. Neurosurg.* **90**, 258–264 (1999). 9950496 10.3171/jns.1999.90.2.0258

[31] Tan, C. & Scotting, P. J. Stem cell research points the way to the cell of origin for intracranial germ cell tumours. *J. Pathol.* **229**, 4–11 (2013). 22926997 10.1002/path.4098

[32] Manoranjan, B., Garg, N., Bakhshinyan, D. & Singh, S. K. The role of stem cells in pediatric central nervous system malignancies. *Adv. Exp. Med. Biol.* **853**, 49–68 (2015). 25895707 10.1007/978-3-319-16537-0_4

[33] Oosterhuis, J. W. & Looijenga, L. H. J. Human germ cell tumours from a developmental perspective. *Nat. Rev. Cancer* **19**, 522–537 (2019). 31413324 10.1038/s41568-019-0178-9

[34] Vaca, A. M. et al. The expansion of adult stem/progenitor cells and their marker expression fluctuations are linked with pituitary plastic adaptation during gestation and lactancy, American journal of physiology. *Endocrinol. Metabolism* **311**, E367-379 (2016). 10.1152/ajpendo.00077.2016 27302752

[35] Guido, C. B. et al. Changes of stem cell niche during experimental pituitary tumor development. *J. Neuroendocrinol.* **33**, e13051 (2021). 34708474 10.1111/jne.13051

[36] Lee, E. et al. Treatment outcomes and prognostic factors of intracranial germ cell tumors: A single institution retrospective study. *Brain Tumor Res. Treat.* **13**, 45–52 (2025). 40347126 10.14791/btrt.2024.0045 PMC12070076

[37] J. Chernoff, The two-hit theory hits 50, Molecular Biology of the Cell, 32 (2021) rt1. 10.1091/mbc.E21-08-0407 PMC8694077 34735271

[38] Zhang, C.-C., Feng, H.-R., Zhu, J. & Hong, W.-F. Application of spatial and single-cell omics in tumor immunotherapy biomarkers. *LabMed Discov.* **2**, 100076 (2025).

