# Supplementary figures and images for: Primordial germ cell-like cells residing in the pituitary may serve as the origin of intracranial germ cell tumors
Source: Sci Rep. 2026 Feb 3;16:7086. doi: 10.1038/s41598-026-38060-2 (PMC12920747; doi:10.1038/s41598-026-38060-2)

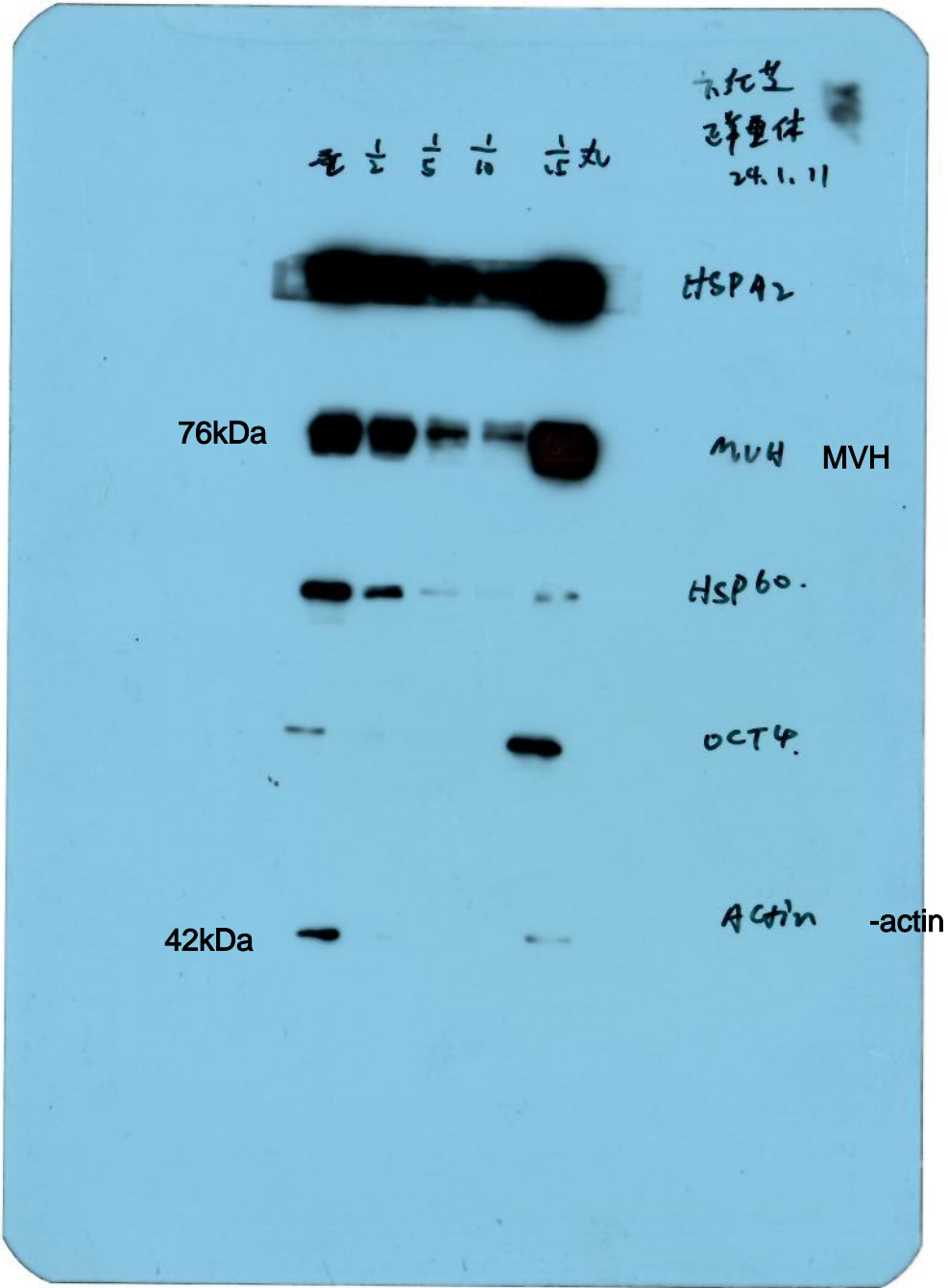

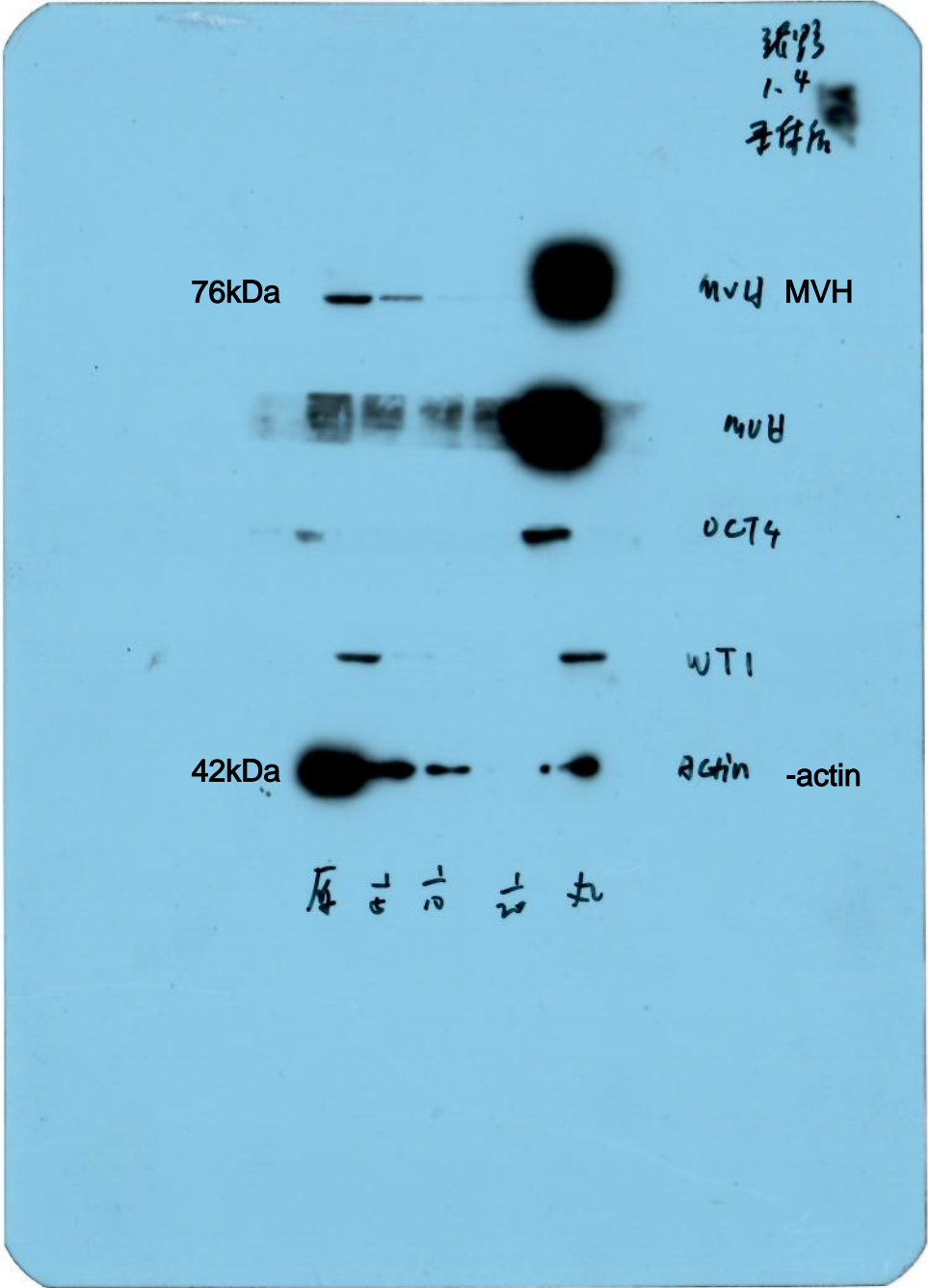

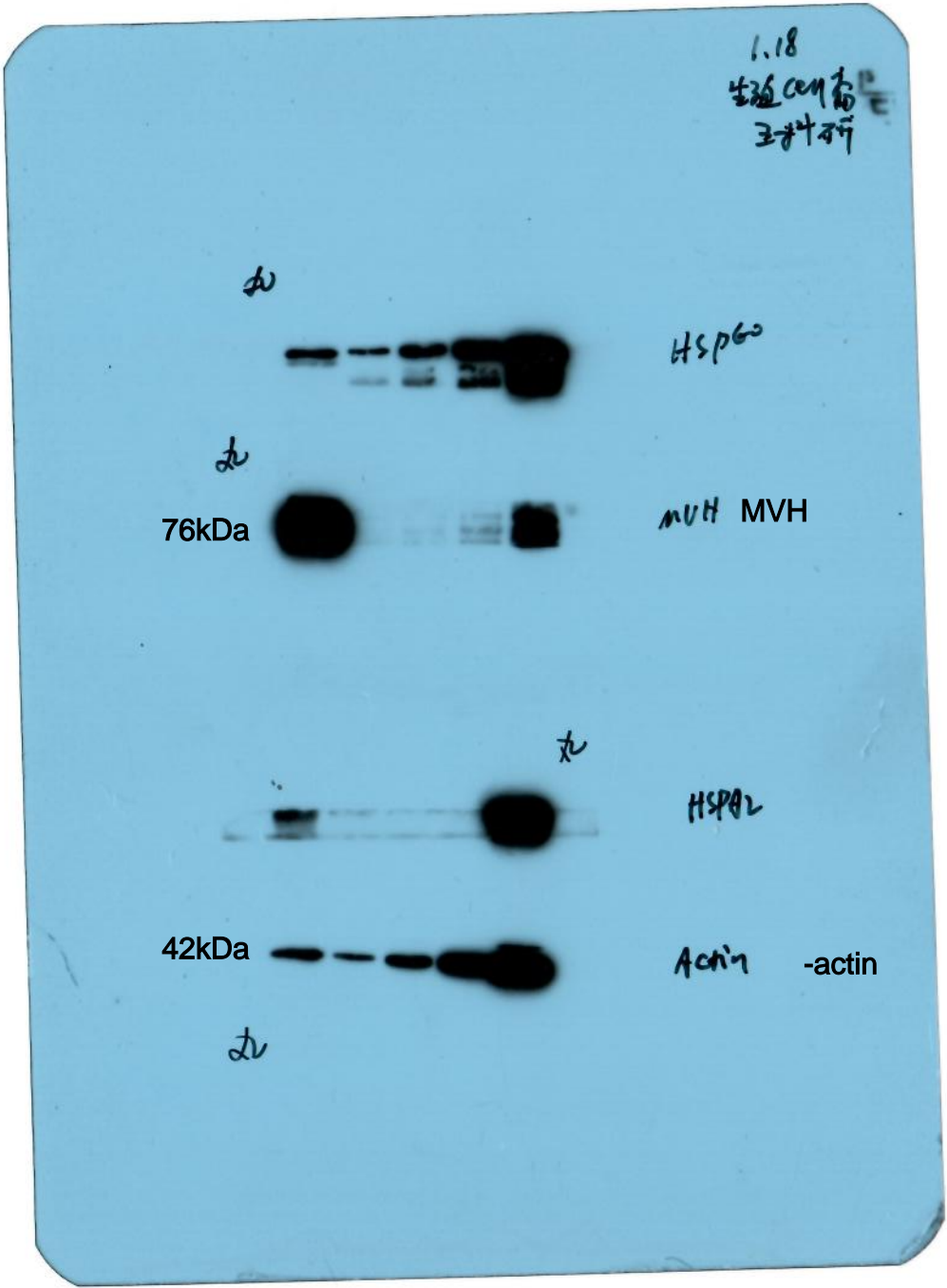

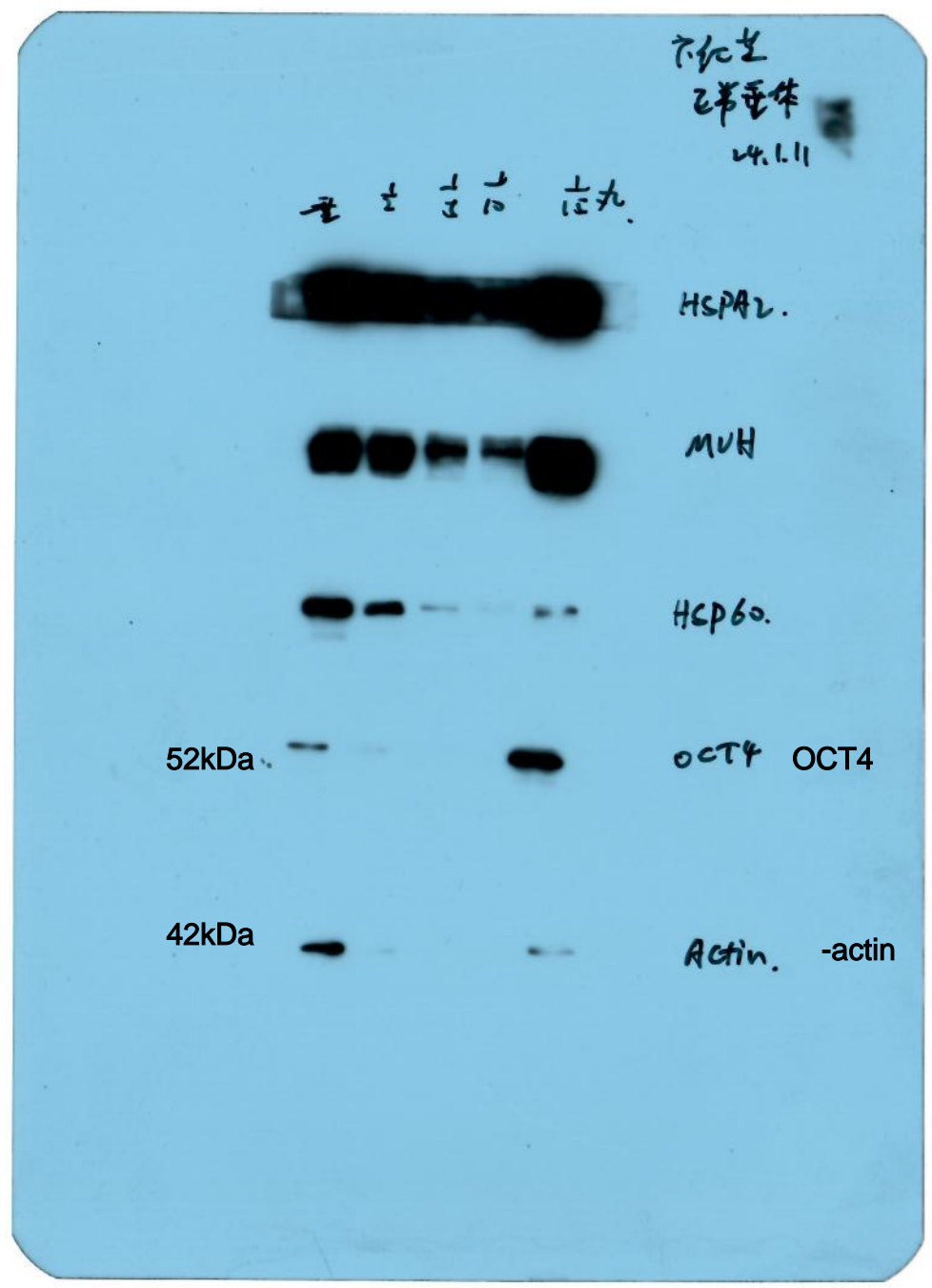

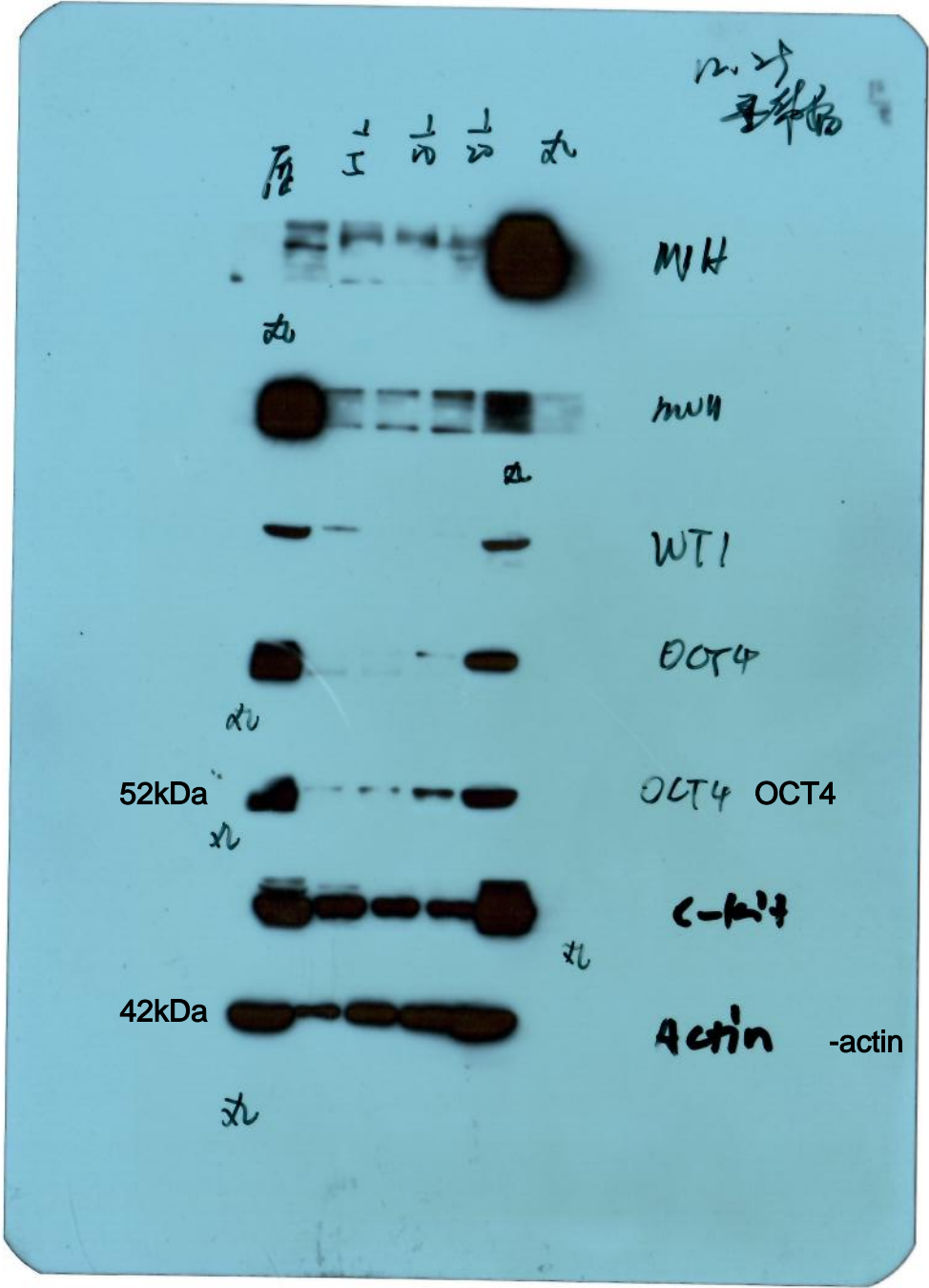

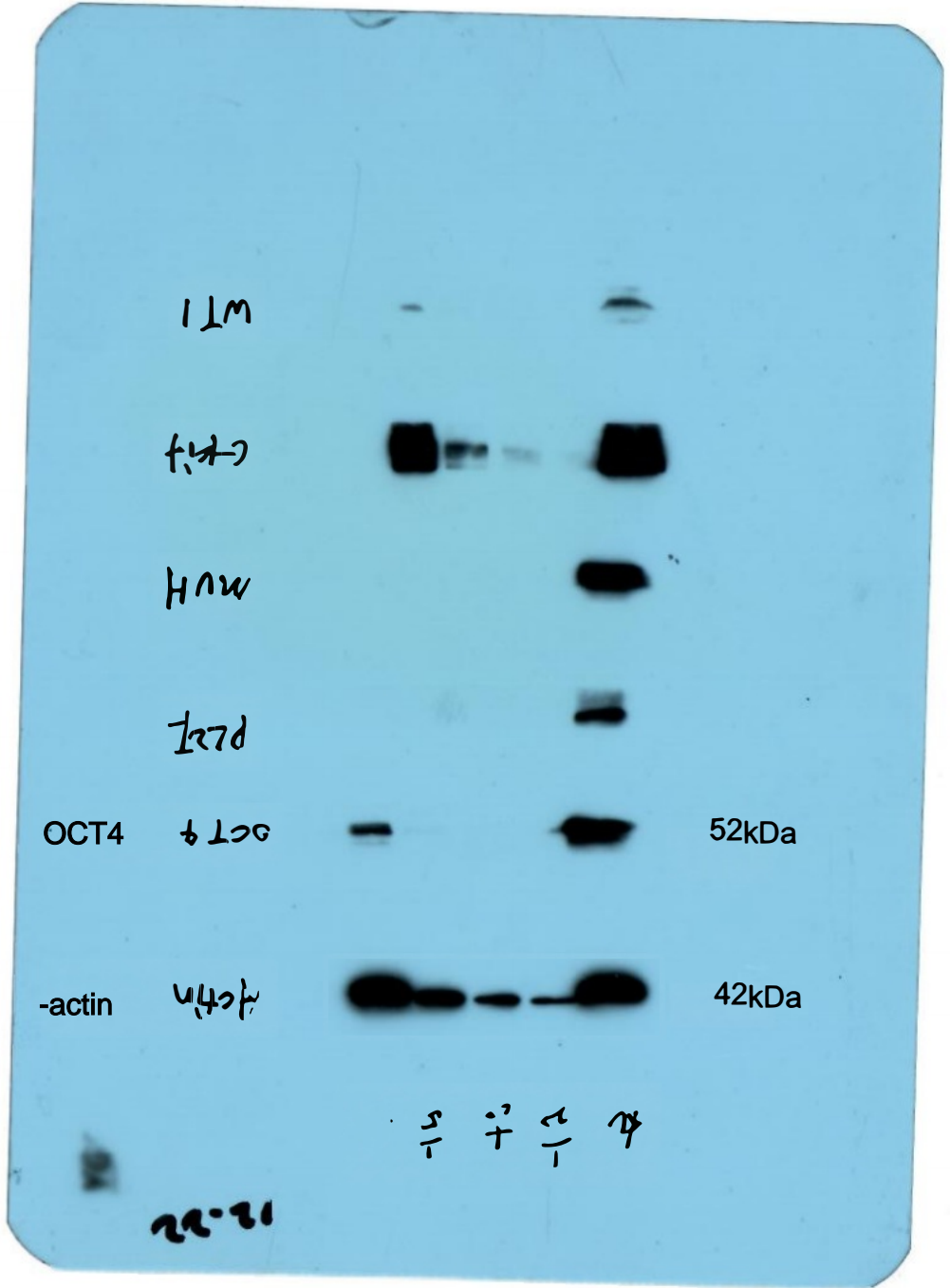

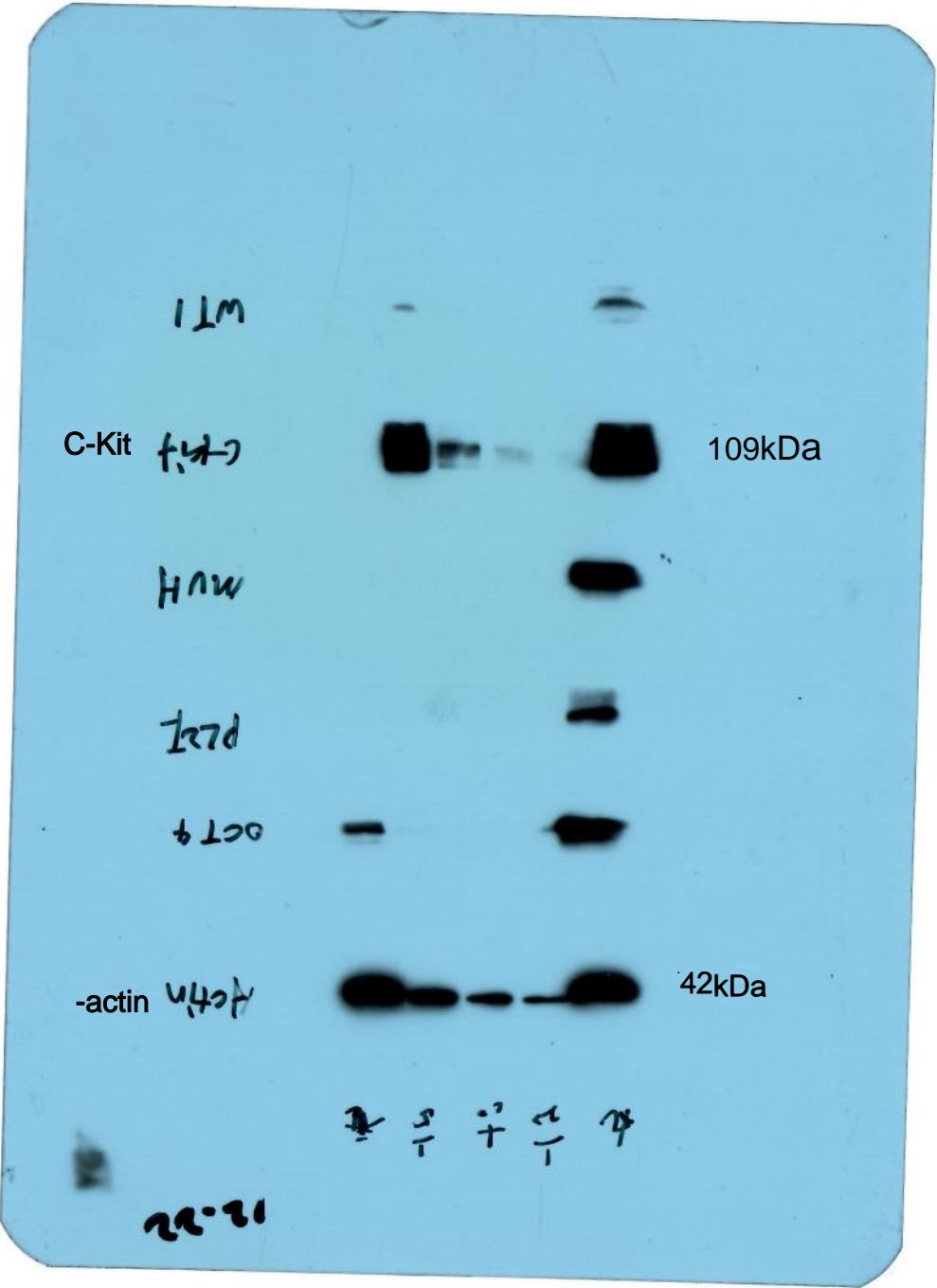

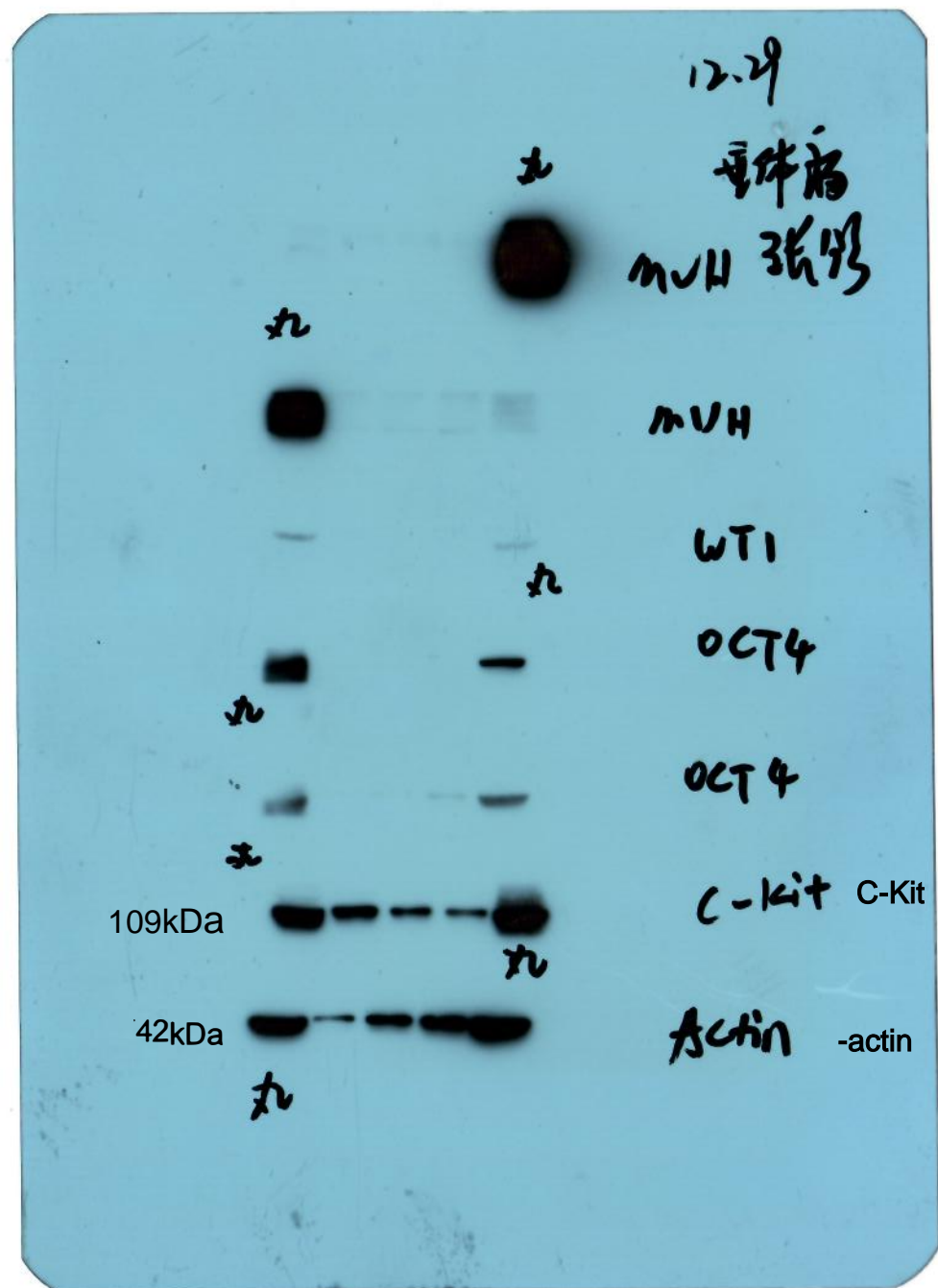

C-Kit-GCT

C-Kit 1.4-7

109kDa

-actin 1.4-7

42kDa

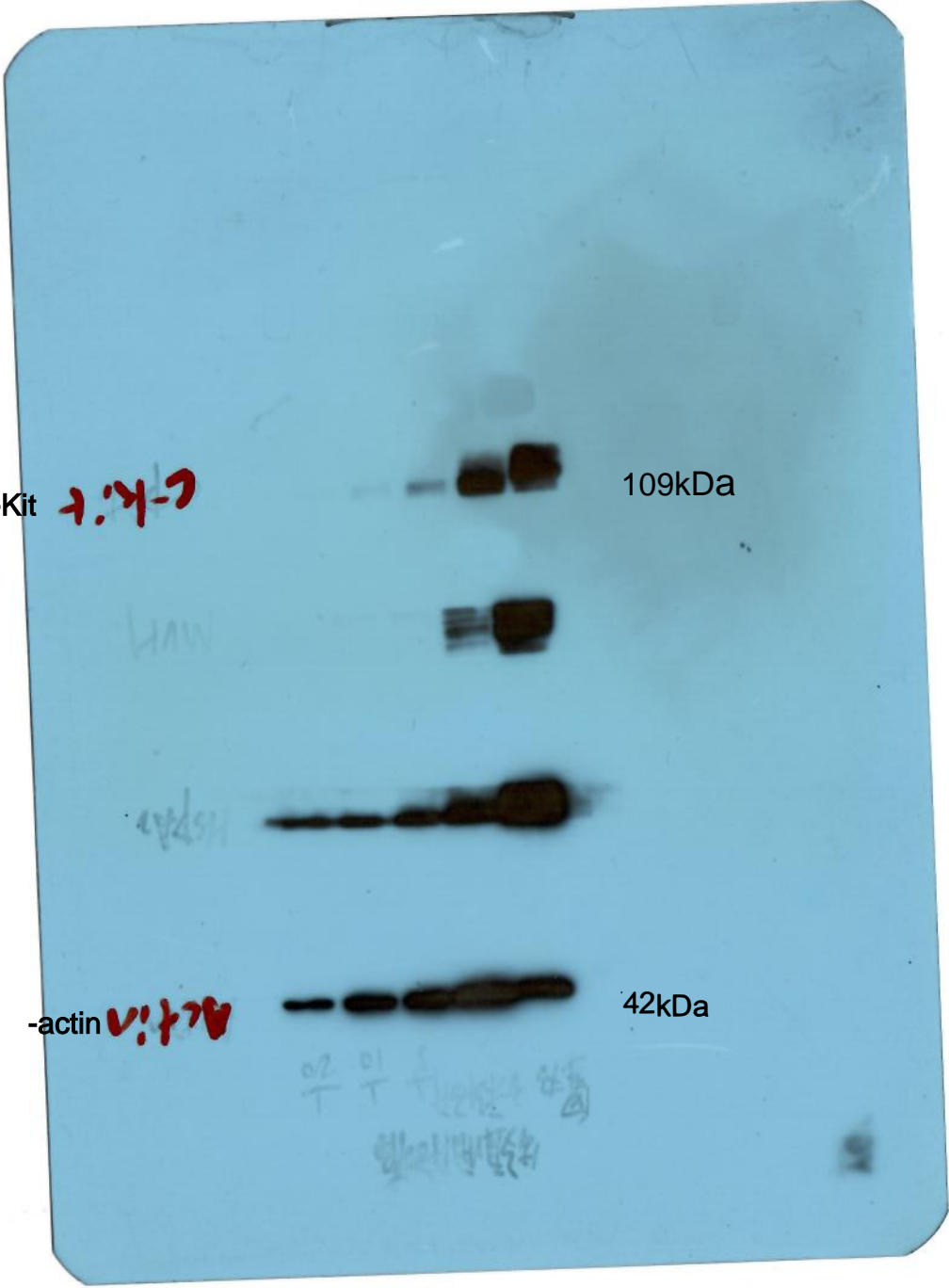

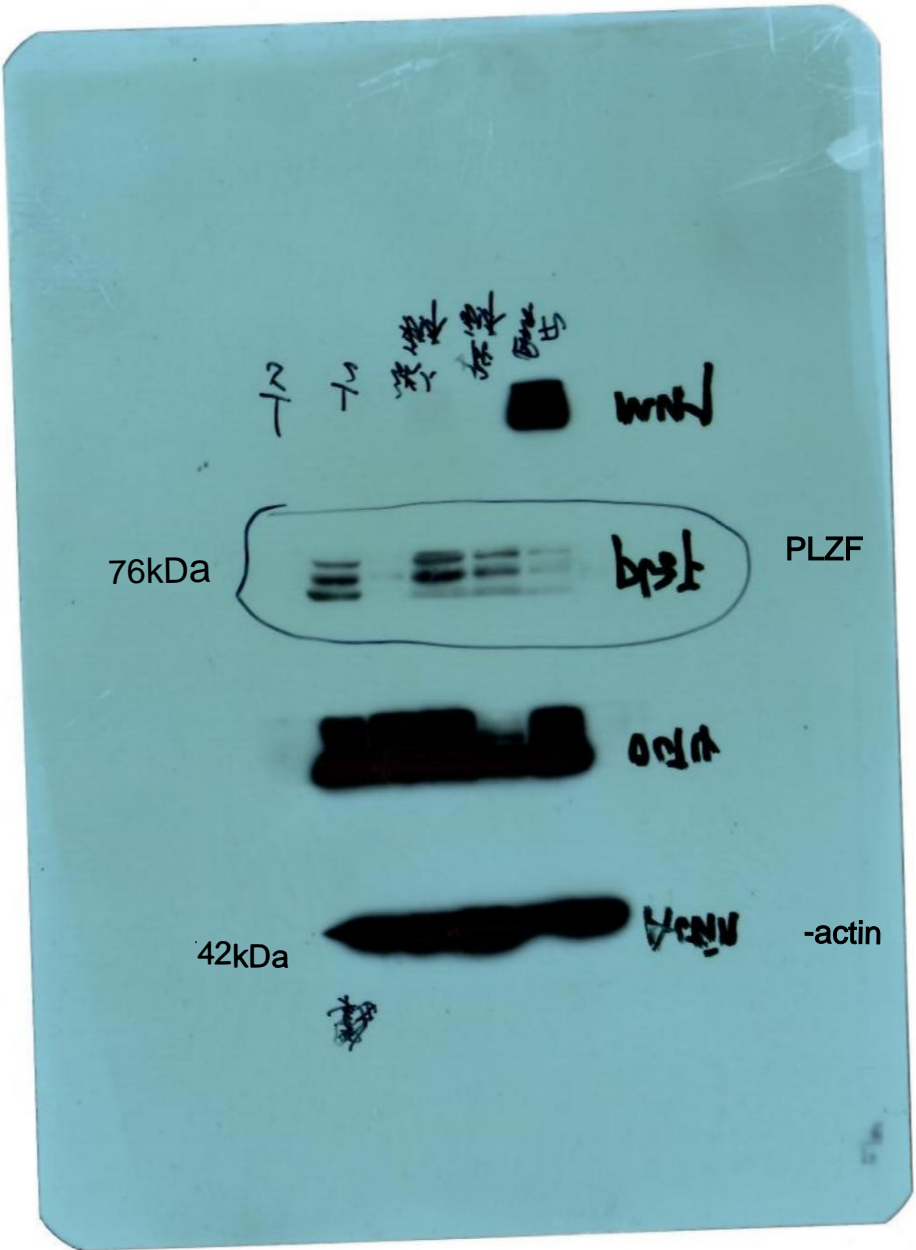

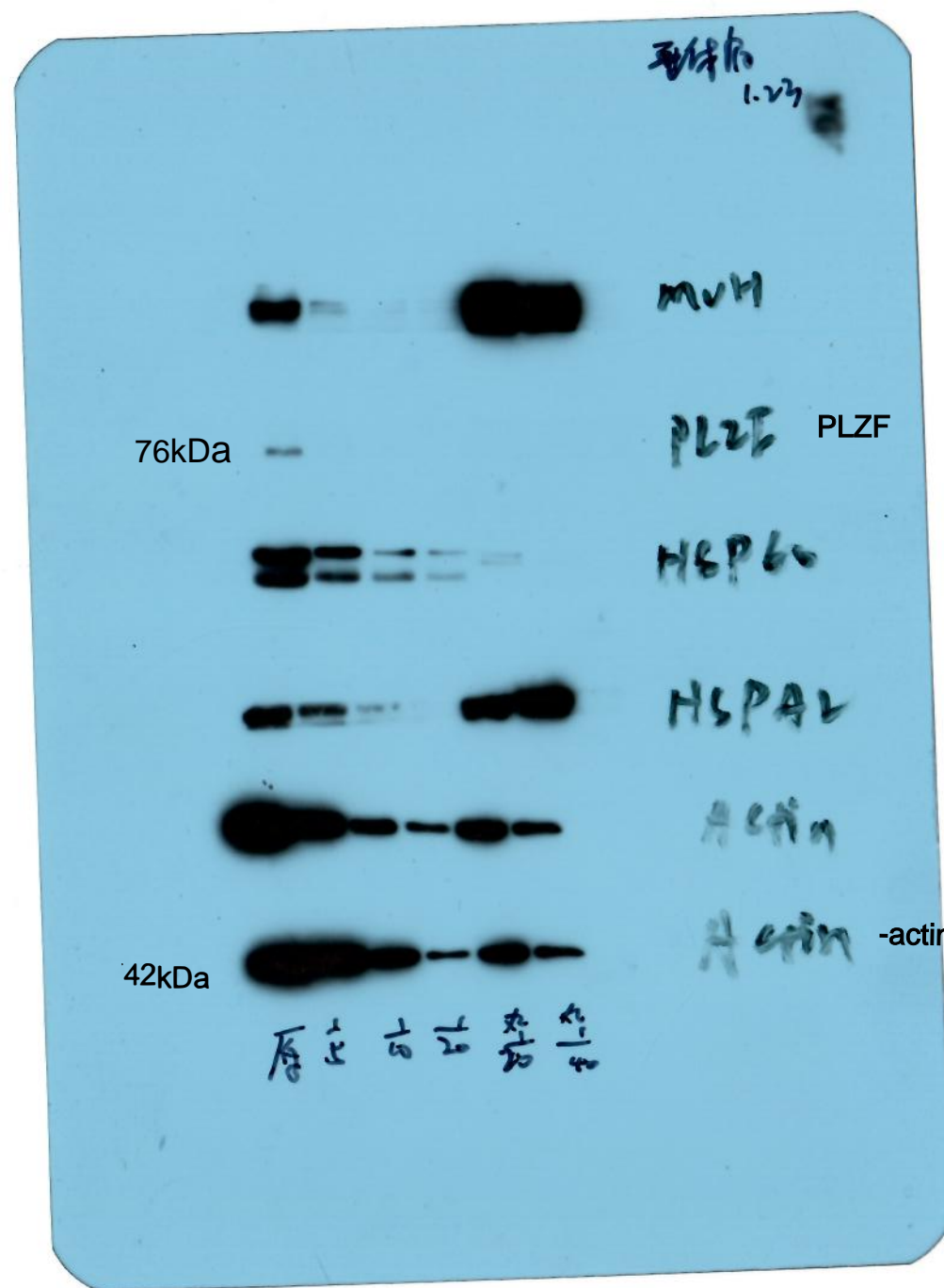

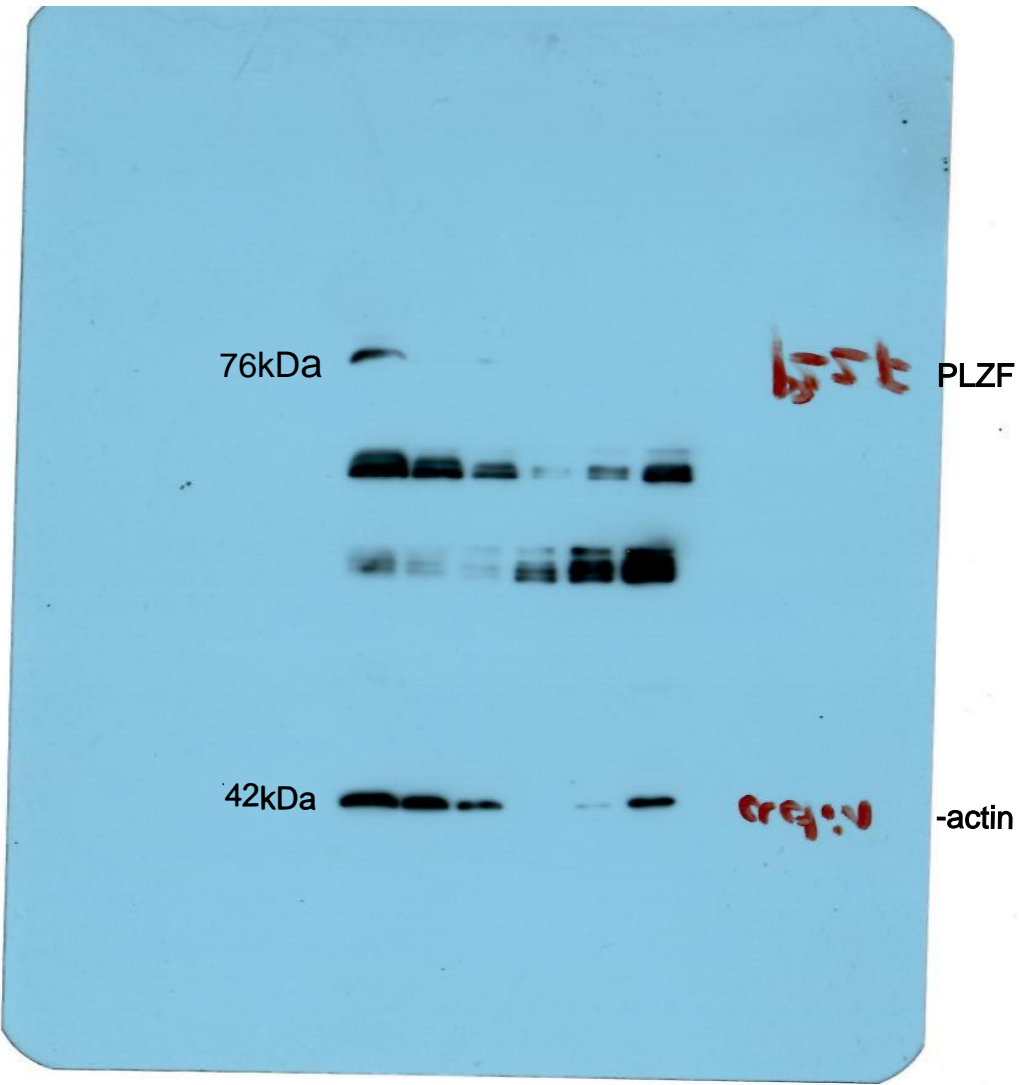

Supplement: Supplementary file 1 — Supplementary Material 1 [file 41598_2026_38060_MOESM1_ESM.pdf]
