# Supplementary material for: Primordial germ cell-like cells residing in the pituitary may serve as the origin of intracranial germ cell tumors
Source: Sci Rep. 2026 Feb 3;16:7086. doi: 10.1038/s41598-026-38060-2 (PMC12920747; doi:10.1038/s41598-026-38060-2)
Supplement: Supplementary file 2 — Supplementary Material 2 [file 41598_2026_38060_MOESM2_ESM.docx]

**Supplementary Table S1.** Clinicopathological characteristics and immunohistochemical profiles of specimens included in this study

| **Case ID** | **Specimen category** | **Pathological diagnosis** | **PitNET subtype** | **Anatomical site** | **Age**  **(years)** | **Sex**  **（M/F）** | **Key immunohistochemical profile** |
| --- | --- | --- | --- | --- | --- | --- | --- |
| P-01 | Normal pituitary | Non-neoplastic pituitary tissue | NA | Pituitary gland | 35 | M | NA |
| P-02 | Pituitary tumor | PitNET | Somatotroph PitNET | Sellar region | 50 | F | **Lineage/hormones:** GH(+), PRL(+), ACTH(−), TSH(−), FSH(−). Transcription factors: PIT-1(+), T-PIT(−), SF-1(−). Proliferation/tumor suppressor: Ki-67 index = 1%, p53(+). Other markers: CAM5.2(+), ER(+), SSTR2 (3+), MGMT(+), CgA(+), IH(−). |
| P-03 | iGCT | Germinoma | NA | Sellar region | 8 | F | **Germ cell/pluripotency markers:** OCT4 (OCT3/4)(+), SALL4(+), SOX2(+), CD117/KIT(+). Epithelial/other markers: AE1/AE3(−), EMA(−), CgA(−). Tumor markers: AFP(−), CD30(Ki-1)(−). Proliferation: Ki-67 index = 45%. |
